# Optimizing TACE for Hepatocellular Carcinoma: The Impact of Intra-Arterial Contrast Enhanced Ultrasound

**DOI:** 10.3390/diagnostics15111380

**Published:** 2025-05-29

**Authors:** Linda Galasso, Jacopo Iaccarino, Giorgio Esposto, Gabriele Giansanti, Irene Mignini, Raffaele Borriello, Gianpaolo Vidili, Antonio Gasbarrini, Maria Elena Ainora, Maria Assunta Zocco

**Affiliations:** 1 CEMAD Digestive Disease Center, Fondazione Policlinico Universitario Agostino Gemelli IRCCS, Catholic University of Rome, 00168 Rome, Italy; linda.galasso@guest.policlinicogemelli.it (L.G.); jacopo.iaccarino01@icatt.it (J.I.); giorgio.esposto@guest.policlinicogemelli.it (G.E.); gabriele.giansanti@guest.policlinicogemelli.it (G.G.); irene.mignini@guest.policlinicogemelli.it (I.M.); raffaele.borriello@unicatt.it (R.B.); antonio.gasbarrini@unicatt.it (A.G.); mariaassunta.zocco@policlinicogemelli.it (M.A.Z.); 2 Department of Medical, Surgical and Experimental Sciences, University of Sassari, 07100 Sassari, Italy; gianpaolovidili@uniss.it

**Keywords:** transarterial chemoembolization (TACE), hepatocellular carcinoma (HCC), intra-arterial contrast-enhanced ultrasound (IA CEUS)

## Abstract

Transarterial chemoembolization (TACE) is a well-established treatment for intermediate-stage hepatocellular carcinoma (HCC), shown through randomized trials to improve survival compared to supportive care in patients with large, unresectable tumors who are not candidates for liver transplantation or local ablation. As the most commonly used transarterial intervention, TACE is also employed to downstage advanced HCC, allowing certain patients to become eligible for orthotopic liver transplantation under the Milan criteria. Despite its widespread use, variability in therapeutic outcomes highlights the need for improved procedural guidance. Recent advancements in intra-arterial contrast-enhanced ultrasound (IA CEUS) offer new opportunities to enhance TACE precision with real-time imaging that provides superior visualization of tumor vasculature and chemoembolic agent distribution. This review explores the role of IA CEUS in refining TACE for HCC, emphasizing its potential to increase intraprocedural accuracy and reduce the risk of incomplete tumor embolization. The enhanced spatial resolution of IA CEUS enables real-time tracking of embolic agent dispersion within tumor vessels, which could improve therapeutic efficacy by ensuring complete tumor targeting and minimizing non-target embolization. Additionally, IA CEUS may decrease procedural complications by allowing dynamic adjustment of embolic delivery based on real-time imaging feedback. By reviewing existing evidence on IA CEUS applications in TACE, this article highlights the modality’s potential to transform treatment protocols, improve outcomes, and expand the patient population eligible for TACE.

## 1. Introduction

Hepatocellular carcinoma (HCC) is the most prevalent primary liver cancer and a leading cause of cancer-related deaths worldwide [1]. It typically arises in individuals with long-standing liver conditions, particularly cirrhosis associated with viral hepatitis (hepatitis B and C), metabolic dysfunction-associated steatotic liver disease (MASLD), and alcohol-related liver disease (ALD) [2,3]. The rising prevalence of HCC highlights the critical need for enhanced diagnostic and treatment approaches [4]. Due to its asymptomatic nature in the early stages, HCC is often diagnosed at an advanced phase, complicating early detection and treatment. The liver’s intricate structure and the aggressive behavior of HCC further challenge disease management [5]. Therefore, therapeutic approaches must be carefully tailored, considering both liver function and tumor extent, with a focus on maximizing survival and maintaining quality of life [6]. HCC management relies on staging systems such as the Barcelona Clinic Liver Cancer (BCLC) classification, which aids in selecting appropriate treatment options based on tumor size, liver function (Child–Pugh score), and overall patient health [7]. Patients with early-stage disease may be candidates for curative treatments, such as liver transplantation, surgical resection, or ablation [7,8]. However, many patients are diagnosed with intermediate-stage HCC, where curative treatments are not an option, making locoregional therapies like transarterial chemoembolization (TACE) an essential part of the treatment strategy [9]. TACE involves delivering chemotherapy directly to the tumor through the hepatic artery, followed by embolization to cut off its blood supply, inducing tumor ischemia and necrosis [10]. This technique takes advantage of the liver’s vascular structure, as HCC tumors primarily receive blood from the hepatic artery, whereas normal liver tissue is mainly supplied by the portal vein [11]. By directly targeting the tumor, TACE enhances chemotherapy effectiveness while minimizing systemic side effects. The embolization process further strengthens the treatment by depriving cancerous cells of oxygen and nutrients, leading to cell death [10]. TACE is a cornerstone therapy for intermediate-stage HCC, particularly for patients classified under BCLC stage B, characterized by multiple tumors or vascular invasion while maintaining liver function [9]. Studies have demonstrated that TACE can effectively reduce tumor size, extend survival, and serve as a bridge to liver transplantation or surgical resection [12,13,14]. Additionally, it can be repeated for patients experiencing tumor progression, making it a valuable option for ongoing disease control [9]. Despite its benefits, TACE outcomes depend on factors such as tumor characteristics, vascular involvement, and liver function. Advancements in embolic materials and imaging techniques have refined the procedure. Traditional TACE, using lipiodol and standard embolic agents, has evolved with the introduction of drug-eluting bead (DEB) TACE, which allows for controlled chemotherapy release while simultaneously blocking blood flow, enhancing drug efficacy and reducing side effects [15]. Challenges with TACE include variable tumor responses, recurrence risk, and the potential for liver dysfunction following repeated treatments. Effective patient selection and continuous monitoring are essential to optimize outcomes [16,17]. Innovations such as contrast-enhanced ultrasound (CEUS) have improved tumor localization, procedural accuracy, and real-time evaluation of treatment success, reduced incomplete embolization, and enhanced the targeting of small or multiple tumors [18,19].

This review aims to explore the role of CEUS in optimizing HCC treatment through TACE, emphasizing its potential to improve precision, efficacy, and overall patient outcomes.

## 2. Challenges in Traditional TACE Procedure

Conventional transarterial chemoembolization (c-TACE), in which cytotoxic drugs are delivered to the tumor using lipiodol, was established based on two randomized controlled trials (RCTs) that demonstrated a significant overall survival benefit [20,21]. Based on these results, TACE became the standard of care for intermediate-stage HCC (BCLC stage B) in patients without hepatic decompensation or cancer-related symptoms [22]. A systematic review of 101 studies (10,108 patients) confirmed the efficacy and safety of c-TACE, showing a median survival of 19.4 months (95% CI 16.2–22.6) and overall survival rates of 70.3% at 1 year, 40.4% at 3 years, and 32.4% at 5 years.

The most common adverse event was postembolization syndrome (PES) characterized by symptoms such as fever, abdominal pain, nausea, and elevated liver enzymes due to tumor ischemia and necrosis occurring in 4.8% of patients, while the mortality rate was 0.6%, primarily due to acute liver failure [23]. In addition to PES, there are more serious complications such as biloma and liver abscess that can occur. A biloma refers to a collection of bile outside the biliary system, often resulting from bile duct injury or leakage during the embolization procedure. A liver abscess, in contrast, is a localized infection that can arise from tissue ischemia and necrosis caused by TACE, which increases the risk of bacterial or fungal infection. Both conditions highlight the importance of careful monitoring and management following TACE [24,25].

The introduction of drug-eluting bead TACE (DEB-TACE) aimed to improve tumor drug delivery and reduce systemic exposure. These outcomes were explored in both the PRECISION V study, the first RCT comparing c-TACE and DEB-TACE [26], and in a real-world study on 471 patients [27] with promising results. Further improvements in disease control rates were then achieved with advances in superselective TACE, which delivers drugs to a sub-segmental hepatic artery [28]. Still nowadays, the clinical superiority of DEB-TACE over c-TACE remains debated, as no RCT has confirmed a significant survival benefit [29].

In Table 1, the actual evidence on TACE’s efficacy and safety is summarized.

## 3. Evaluation of Treatment’s Response

Serial imaging is essential for monitoring HCC after locoregional treatments, enabling the evaluation of therapeutic response and early detection of residual or recurrent tumors. The primary imaging modalities for assessing responses after TACE and other liver therapies are CT and MRI [30]. Radiological follow-up is typically scheduled for 1 and 3 months, then every 3 to 6 months, with early response at 1 month strongly correlated with overall survival rates [31]. The mRECIST criteria, established in 2010, are the standard for evaluating response to locoregional therapies and systemic treatments for HCC [32]. Post-treatment imaging features include a hypoattenuating area on CT and hypointense regions on T2WI, indicating necrosis, while enhancing foci suggest viable tissue [33]. Residual tumors typically appear as thick (>5 mm) nodular areas with arterial phase hyperenhancement (APHE) and washout in the portal/delayed venous phases [34]. MRI, with its higher sensitivity, is preferred over CT for detecting HCC recurrence [35] and can be enhanced with diffusion-weighted imaging (DWI) for more accurate assessment [36]. However, both CT and MRI have limitations, such as radiation exposure from CT and beam hardening artifacts from Lipiodol^®^ used in cTACE [37]. Lipiodol^®^ correlates with complete necrosis, whereas DEB-TACE facilitates easier assessment due to the absence of iodized oil [38]. Despite these drawbacks, CT remains popular due to its availability, lower costs, and utility when MRI is contraindicated or less effective, such as in patients with ascites [39].

## 4. CEUS: Enhancing Imaging Precision in HCC

CEUS with second-generation contrast agents (SonoVue) has gained particular attention in this setting since the need to explore alternative imaging modalities for TACE therapeutic assessment is still unsolved. The European Federation of Societies for Ultrasound in Medicine and Biology (EFSUMB) highlights the role of CEUS in detecting and monitoring tumor response in focal liver lesions after locoregional and systemic HCC treatments [40].

### 4.1. Microvascular Imaging and Safety of CEUS

A significant advantage of CEUS is its ability to avoid the risks of nephrotoxicity associated with contrast agents used in CT and MRI. CT often uses iodinated contrast agents, which can cause contrast-induced nephropathy (CIN), particularly in patients with pre-existing renal conditions. Similarly, gadolinium-based contrast agents in MRI can lead to nephrogenic systemic fibrosis (NSF) in individuals with renal impairment. In contrast, CEUS employs microbubbles that are not nephrotoxic, as they are cleared by the lungs, making it a safer option for patients with compromised renal function. Additionally, CEUS eliminates the risk of ionizing radiation exposure, which is inherent in CT and fluoroscopy. CEUS, however, provides high-quality, real-time imaging without ionizing radiation, offering a safer alternative for repeated imaging, especially in vulnerable patient populations. Moreover, Lipiodol^®^ obscuration, a challenge in MRI imaging post-TACE, can significantly reduce image clarity due to the dense iodine content of Lipiodol^®^ that accumulates in the embolized tumor. This accumulation can obscure MRI images, particularly in T1-weighted sequences, making it difficult to distinguish between viable and necrotic tumor tissue. Although Lipiodol^®^ is visible in CT, it does not offer sufficient detail for assessing post-treatment perfusion or detecting subtle changes in tumor vascularity. CEUS circumvents these challenges entirely, providing clear and accurate imaging of tumor perfusion without interference from Lipiodol^®^ deposits, making it an especially valuable tool for post-TACE monitoring [40,41].

Microvascular characteristics in CEUS are assessed through the behavior of intravenously injected microbubbles, which serve as contrast agents ranging from 1 to 10 μm in diameter [42]. These microbubbles are small enough to pass through capillaries but large enough to generate strong acoustic signals, with their concentration directly influencing the intensity of the ultrasound signal. Unlike conventional B-mode ultrasound, which struggles to detect microvascular flow due to limited sensitivity and tissue interference, CEUS employs second-harmonic or nonlinear imaging modes that exploit the nonlinear oscillation of microbubbles to generate enhanced harmonic signals, particularly at the second-harmonic frequency. This allows for more accurate visualization of blood flow in small vessels with high spatial and temporal resolution, improving the assessment of tissue perfusion and vascularization [40,42]. To optimize microbubble visibility while maintaining their stability, CEUS uses carefully adjusted parameters such as a low mechanical index (MI) between 0.05 and 0.2 to minimize bubble destruction, allowing continuous real-time imaging of microvascular flow [40,43]. The ultrasound frequency is generally set between 1 and 7 MHz, balancing penetration depth with spatial resolution, and frame rates of 10 to 25 frames per second are used to optimize the temporal resolution and signal-to-noise ratio. CEUS relies on contrast-specific imaging modes like pulse inversion or amplitude modulation, often in second-harmonic or nonlinear modes, to selectively enhance microbubble signals and suppress background tissue echoes [40,44]. When microbubbles are injected into arteries, they remain stable under normal physiological conditions, as the arterial environment does not typically cause breakdown or fusion. Instead, microbubble destruction is primarily influenced by ultrasound settings, with high MI levels leading to collapse through inertial cavitation, while low MI settings preserve bubble integrity. Fusion of microbubbles is not a common occurrence in vivo due to the design of the shell materials, which ensure individual bubble stability during circulation. Thus, arterial injection of CEUS microbubbles remains safe and effective for imaging, without significant disruption to the bubbles themselves [45].

### 4.2. CEUS Accuracy in Post-TACE Residual Tumor Detection

Residual tumors show internal or peripheral enhancement, while completely treated HCC exhibits smooth margins with no internal flow [46]. CEUS is highly accurate in assessing intratumoral vascularity, detecting even small viable tissue through real-time microvessel perfusion [47]. Advancements in CEUS have improved its ability to assess treatment response. The new ACR CEUS Non-radiation TRA LI-RADS v2024 criteria are reliable for detecting viable tumors in TACE-treated lesions [48]. CEUS is more sensitive and accurate for early HCC response, with some studies demonstrating its effectiveness as early as one day post-TACE [49]. Takizawa et al. showed that CEUS was comparable to contrast-enhanced CT (CECT) at 4 weeks after TACE, enabling earlier detection of recurrences [50]. S.B. Paul et al. found that CEUS had a sensitivity of 94% and a specificity of 100% in detecting residual disease compared to MRI [51]. A meta-analysis of 421 patients confirmed CEUS’s superior sensitivity and negative predictive value compared to CECT [52]. Pilot data suggest CEUS is comparable to CT and MRI for small lesions [18]. Despite these advantages, CEUS has some limitations, such as higher intra- and inter-reader variability due to operator dependence [53] and challenges in visualization in patients with obesity, cirrhosis, or tumors near the diaphragm [54]. Factors like lesion multiplicity, deep location, hypoenhancement, and diffuse tumor growth can also affect CEUS efficiency after TACE [55].

Several advanced CEUS techniques have been developed to address its limitations. Dynamic contrast-enhanced ultrasound (DCE-US) quantifies tissue perfusion over time, generating time–intensity curves to assess microvascular blood flow with parameters like peak enhancement (PE), area under the curve (AUC), wash-in rate (WiR), rise time (RT), time to peak (TTP), and mean transit time (mTT), useful for evaluating neoplastic tissue and therapy response [56]. DCE-US has been explored in a few post-TACE success studies. For example, Uller et al. showed significant devascularization following DEB-TACE, with the AUC ratio between HCC and liver decreasing significantly (AUC ratio HCC/liver: 2.6 ± 1.5 before, 0.45 ± 0.25 after) [57]. Cao et al. found significant reductions in AUC and peak intensity in responders compared to non-responders in 40 patients undergoing TACE [58]. Nam et al. demonstrated greater declines in perfusion parameters in responders using 2D and 3D CEUS at baseline, 1–2 weeks, and 1 month post-TACE [59]. A multicenter trial compared the diagnostic performance of 2D and 3D CEUS in detecting residual viable HCC post-TACE and found both had higher sensitivity (91% for 2D CEUS and 89% for 3D CEUS) than CE-MRI (68%) and CT (58%) (*p* < 0.001). No significant differences were observed between the two CEUS modalities [60].

Table 2 provides a comparative overview of imaging modalities used for post-TACE monitoring in patients with hepatocellular carcinoma (HCC).

## 5. IA-CEUS: Transforming Real-Time Tumor Targeting

**Figure 1:**
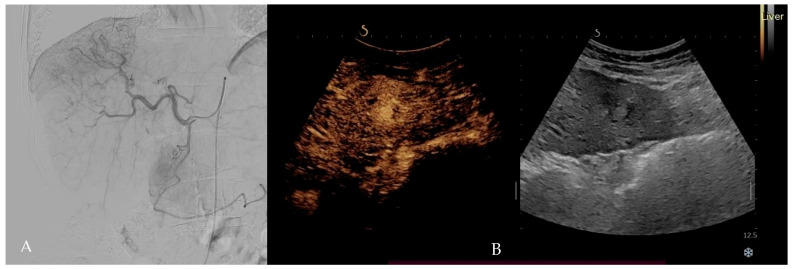
IA-CEUS—intra-arterial contrast-enhanced ultrasound of an HCC nodule of VII liver segment. (**A**): Angiographic phase; (**B**): arterial phase (20 secs) of IA-CEUS.

Intra-arterial contrast-enhanced ultrasound (IA-CEUS) is a novel technique in which the microbubble contrast agent is injected directly into the artery during catheter-based arteriography, allowing for a more selective evaluation of the arterial supply to the tumor [61] (Figure 1). In IA-CEUS, the image analysis is essential for evaluating tumor vascularity before treatment, guiding catheter placement during the procedure, and assessing therapeutic response afterward. The analysis includes both qualitative and quantitative assessments. Qualitatively, radiologists assess the lesion’s enhancement pattern, including the timing and intensity of contrast uptake during arterial, portal venous, and late phases, focusing on features such as rapid wash-in and wash-out that are characteristic of hepatocellular carcinoma. Quantitatively, time–intensity curves (TICs) may be generated from selected regions of interest to measure parameters such as time to peak, peak intensity, wash-in slope, and area under the curve, which provide objective indicators of perfusion. CEUS during TACE is typically performed by an interventional radiologist or a trained sonographer under the supervision of a radiologist, ensuring real-time imaging guidance to optimize treatment delivery and monitor therapeutic outcomes [40,62].

IA-CEUS helps to visualize the real-time changes in the microcirculation of the HCC lesion during and after locoregional treatment, allowing the evaluation of the feeding artery with its intratumoral branches and the local venous drainage surrounding the tumor.

Among the first to explore this new technique were Moschouris H et al., who conducted a pilot study in 2011 involving 17 hepatic lesions treated with TACE. The authors used IA-CEUS to identify the feeding artery and then proceeded to TACE, increasing the accuracy of superselective embolization. This approach allowed for the exclusion of arteries previously thought to supply the lesion and enabled the identification of the superselective vessel of the lesion, whereas standard angiography had failed [55].

Later, in 2017, Lekht I et al. described a case series of three patients with radiological findings of HCC previously treated with conventional DEB-TACE, in which IA-CEUS initially enabled lesion visualization, outperforming superselective angiography. Moreover, IA-CEUS guidance allowed for the observation of the residual blood flow origin of these lesions, optimizing the intervention by identifying the superselective branch feeding the tumor and enabling targeted embolization. Follow-up imaging confirmed the absence of residual tumor, indicating complete treatment [62].

These advantages were achieved without any adverse side effects, but they needed to be confirmed on larger cohorts due to the paucity of patients enrolled.

The feasibility of IA-CEUS in both TACE and DEB-TACE was then studied by Fei X et al. [63], Shiozawa K et al. [64] and Bo et al. [65].

Fei X. et al. enrolled 39 patients with a total of 51 HCC lesions subjected to conventional TACE with IA-CEUS guidance and evaluated the treatment response rate on follow-up with MRI. In this cohort, 43.1% of lesions achieved complete remission and 27.5% showed partial remission, while only 9.8% demonstrated disease progression. The difference in treatment outcomes was associated with two key parameters: the peak value and the thickness of corona enhancement right after the procedure [63]. The therapeutic efficacy of IA-CEUS was also explored by Shiozawa K. et al. in a group of 39 small HCCs (diameter < 50 mm). In these cases, the complete response rate of DEB-TACE is usually low, since details of the tumor vascularity are hard to visualize on digital subtraction angiography (DSA). For example, when two blood vessels are close at the same level in an anteroposterior arrangement, the positional correspondence is hard to identify with DSA. The authors performed DEB-TACE with IA-CEUS guidance, trying to overcome these limits. Each time residual tumor enhancement was judged negative on DSA, IA-CEUS highlighted a residual enhancement in the tumor, allowing DEB-TACE to be repeated. The overall complete response rate at 6 months after treatment (evaluated both by CEUS and CT) was 61.5%, underlining the potential role of IA-CEUS as real-time guidance for improvement of TACE’s efficacy. Importantly, repeated treatment did not result in any instances of severe postembolization syndrome—such as biloma or liver abscess—nor in any adverse events requiring prolonged hospitalization [64].

Indeed, by providing real-time, high-resolution visualization of tumor vasculature and chemoembolic agent distribution, IA-CEUS allows for precise intraprocedural adjustments, minimizing interferences from peripheral tissue enhancement and providing a clearer depiction of tumor washout dynamics. These features may help to predict the risk of local recurrence or metastatic spread and reduce the likelihood of incomplete embolization, ultimately improving therapeutic efficacy. The ability to clearly differentiate tumor tissue from healthy liver parenchyma helps avoid unnecessary embolization of functional liver tissue, thereby reducing the risk of liver dysfunction and preserving hepatic function. In addition, its precision would allow for optimization of drug dosing, potentially reducing the amount of chemotherapeutic agent required while maintaining therapeutic efficacy. However, CEUS-TACE is contraindicated in patients with known hypersensitivity to ultrasound contrast agents, severe cardiopulmonary conditions (such as unstable angina or right-to-left cardiac shunts), or poor acoustic windows that preclude adequate visualization of the target lesion [40]. Additionally, patients with diffuse or infiltrative HCC not amenable to segmental embolization may not be suitable candidates. Careful patient selection ensures optimal imaging outcomes and enhances the effectiveness of CEUS-guided therapy.

Another important advantage of IA-CEUS in TACE is its role in patient selection. While biochemical markers and anatomical factors remain fundamental for determining TACE eligibility—independent of CEUS—IA-CEUS provides additional refinement by evaluating microvascular characteristics and perfusion patterns. This assessment may help to predict treatment response and enables a more personalized therapeutic approach.

Further evidence on the advantages and applicability of IA-CEUS was collected by Bo et al., who enrolled 44 consecutive patients for TACE and performed IA-CEUS before and after the procedure. MRI at 1–3 months after TACE was considered as the reference standard. The multivariate analysis conducted by the authors identified key risk factors for non-response after TACE, including larger lesion diameter, a greater non-enhancing area in superior mesenteric artery (SMA) CEUS compared to preoperative B-mode ultrasound, the presence of corona enhancement in hepatic artery (HA) CEUS postoperatively, and a decrease in corona enhancement thickness (per centimeter) postoperatively. Upon these variables, they developed a logistic predictive model (AUROC of 0.9 and a cross-validation accuracy of 77%) to predict tumor response after TACE by integrating intraprocedural IA-CEUS [65].

Based on this evidence, IA-CEUS marks a significant breakthrough in enhancing the precision and effectiveness of TACE for HCC, though more studies on larger cohorts are needed to confirm its applicability.

Table 3 presents literature studies that report on the diagnostic accuracy of intravenous and intra-arterial CEUS performed during and after TACE.

## 6. Conclusions

As techniques for assessing clinical and radiological response to TACE continue to advance, CEUS and its emerging modalities (DCE-US, 3D-CEUS, IA-CEUS) are expected to play a crucial role in the early evaluation of treatment effectiveness.

IA-CEUS enhances procedural precision, lowers the risk of incomplete embolization, and minimizes unintended injury to healthy liver tissue. Its ability to improve patient selection and predict treatment response further reinforces its value in personalized cancer therapy. Additionally, IA-CEUS is a safe, feasible, and highly effective tool for guiding superselective TACE, leading to better therapeutic outcomes while optimizing drug dosage and preserving liver function. Nonetheless, IA-CEUS also has several limitations, including operator dependence, reduced ultrasound visibility in obese patients, and the lack of standardized imaging protocols, which further hinder its widespread adoption and consistency in clinical practice. Future advancements in IA-CEUS could overcome these challenges by establishing standardized protocols and integrating a multimodal imaging approach for a more comprehensive assessment of HCC recurrence and procedural efficacy. With its potential to refine image-guided liver cancer treatment, IA-CEUS may set a new standard of care in the field.

## Figures and Tables

**Table 1:** Efficacy and safety of TACE.

Study	Design	Patients (n)	Treatment	Results
Llovet JM et al. [20]	Single-institution RCT	112	c-TACE vs. Best Supportive Care	HR 0.47 (95% CI 0.25–0.91; *p* = 0.025); 2-year OS: 63% (TACE) vs. 23% (BSC)
Lo. Chung-Mau et al. [21]	Single-institution RCT	80	c-TACE vs. Best Supportive Care	2-year OS: 31% (TACE) vs. 11% (BSC); RR of death reduction 0.49 (95% CI 0.29–0.81; *p* = 0.006)
Lencioni R. et al. [23]	Meta-analysis	10,108	c-TACE	Median OS: 19.4 months (95% CI 16.2–22.6); 1-year OS: 70.3%; 3-year OS: 40.4%; 5-year OS: 32.4%; PES: 4.8%; mortality: 0.6%
Lammer J. et al. [26]	RCT	201	c-TACE vs. DEB-TACE	OR: 27% vs. 22%; DCR: 63% vs. 52%; no statistical superiority (*p* = 0.11)
Makary MS et al. [27]	Retrospective	471	DEB-TACE	OS: 1-year 64%, 3-year 16.3%, 5-year 2.1%; mean PFS: 6.7 months; 12.5% underwent transplantation

**Table 2:** Comparative evaluation of imaging modalities for post-TACE monitoring in HCC patients.

FOCUS	CEUS	CT	MRI
**Advantages**	- Cost-effective. - No concerns about breath holding or claustrophobia. - No radiation or nephrotoxicity risk [41].	- Widely available. - Lower cost. - Preferred in specific conditions [39].	- Highly sensitive in detecting HCC recurrence [35].
**Limitations**	- Operator-dependent with higher intra- and inter-reader variability [54]. - Visualization challenges (e.g., obesity, cirrhosis, diaphragm location) [55].	- Radiation exposure [37]. - Lipiodol^®^ artifacts hinder accurate interpretation [37].	- Limited sensitivity in patients with ascites [35].
**Sensitivity in** **Tumor Detection**	- High sensitivity (up to 94%) for detecting residual/ recurrent disease, superior to CT [51].	- Moderate sensitivity (50%) for detecting residual disease post-TACE [51].	- Moderate sensitivity (68%) for recurrence [49].
**Follow-Up Protocol**	- 1 and 3 months post-TACE for early detection of recurrence [49].	- Follow-up at 1 and 3 months, then every 3–6 months [31].	- Follow-up at 1 and 3 months, then every 3–6 months [31].
**Evaluation Criteria**	- mRECIST criteria for evaluating treatment response [47].	- mRECIST criteria; hypoattenuation and enhancement patterns for assessment [32,33].	- mRECIST criteria; additional diffusion-weighted imaging (DWI) for atypical features [36].
**Early Detection of** **Viable Tumors**	- Detects viable tumors 1–2 days post-TACE [50].	- Less sensitive for early detection [47].	- Detects recurrence early but less sensitive than CEUS [35].
**Advanced Techniques**	- DCE-US (dynamic contrast-enhanced ultrasound), 3D-CEUS, IA-CEUS [57,61,62].	- No advanced techniques for TACE follow-up [37].	- DWI for atypical post-TACE features [36].
**Post-Treatment Imaging Features**	- Viable tumors show nodular or peripheral enhancement [46].	- Treated areas show hypoattenuation, no enhancement [33].	- Treated areas appear hypointense on T2WI [33].

**Table 3:** Overview of studies reporting CEUS diagnostic performance in the context of TACE.

Study	Design	Patients (n)	Diagnostic Accuracy Post-TACE	Results
Shaw. CM. et al. [46]	Retrospective	54	CEUS vs. RM	Area under the ROC curve (AUC) with CEUS (0.94; 95% confidence interval [CI] 0.88–1.00) vs. gadoxetate disodium-enhanced MR (0.84, 95% CI 0.74–0.93, *p* = 0.0014)
Xia Y. et al. [49]	Prospective	43	CEUS vs. CECT	Detection rate CEUS (58.1%) vs. CECT (39.5%) (*p* < 0.05)
Takizawa K et al. [50]	Prospective	46	CEUS vs. CECT	Detection rate CEUS (95.7%) vs. CECT (78.7%) (*p* < 0.05)
Paul SB. Et al. [51]	Prospective	50	CEUS vs. CECT	Sensitivity: CEUS 94% (34/36; 95% CI: 81–99%); TC 50% (18/36; 95% CI: 33–67%) Specificity: CEUS 100%; TC 100%
Zhong J. et al. [52]	Meta-analysis	421	CEUS vs. CECT	Sensitivity: CEUS 0.97 (95% CI: 0.95–0.99); CECT 0.72 (95% CI: 0.67–0.76) NPV: CEUS 0.90 (0.83–0.95) vs. CECT 0.51 (0.44–0.58)
McGillen KL. et al. [18]	Prospective	26	CEUS vs. CECT/MRI	CEUS: LI-RADS treated 48.7%; LI-RADS viable 61.3% CT/MRI: LI-RADS treated 48.5%; LI-RADS viable 51.5%, *p* = 0.617
Uller W. et al. [57]	Prospective	11	DCE-US (before and after DEB-TACE)	Evaluation of microcirculation in HCC i.v. and i.a. CEUS: reduction of vascularization after bead application was correlated significantly with i.a. and i.v. contrast application (*p* = 0.007) and decreased significantly using TIC analysis (*p* = 0.003)
Cao J. et al. [58]	Prospective	53	2D CEUS and 3D CEUS	Analyzed microperfusional changes before and after TACE: area under the ROC curves for significant ratios and differences of dynamic 3D-CEUS perfusion parameters were higher than those for the corresponding parameters of 2D-CEUS
Nam. K. et al. [59]	Prospective	17	2D CEUS and 3D CEUS vs. MRI	Compared dynamic 2D and 3D CEUS at different time points after TACE: correlation coefficients between 2D and 3D residual tumor estimates in 1–2 weeks post-TACE and the estimates from MRI were 0.73 and 0.94, respectively, while those from 2D and 3D CEUS at 1 month post-TACE were 0.66 and 0.91, respectively.
Savsani E. et al. [60]	Prospective multicenter trial	132	2D CEUS and 3D CEUS vs. CE-MRI and CECT	Sensitivity: 91% for 2D CEUS, 89% for 3D CEUS, CE-MRI 68% and CECT 58% (*p* < 0.001)
Fei X. et al. [63]	Prospective single-center trial	39	IA-CEUS	Maximum cross-sectional area ratio of intratumor perfusion (*p* < 0.001), peak value (*p* < 0.001) and level of corona enhancement (*p* < 0.001) showed correlation with tumor response on CE-MRI
Shiozawa K. et al. [64]	Prospective single-center trial	32	IA-CEUS	Overall CR rate at 6 months 61.5% (24/39).
Bo J. et al. [65]	Case–control single-center study	44	IA-CEUS	Area under the receiver operating characteristic curve (AUROC) of the predictive model 0.904 (95% CI: 0.804, 0.966; *p* < 0.001).

## Data Availability

No new data were created or analyzed in this study. Data sharing is not applicable to this article.

## Abbreviations

The following abbreviations are used in this manuscript:

ACR	American College of Radiology
ALDA	Alcohol-related Liver Disease
APHE	Arterial Phase Hyperenhancement
AUC	Area Under the Curve
AUROC	Area Under the Receiver Operating Characteristic Curve
BCLC	Barcelona Clinic Liver Cancer
CE-MRI	Contrast-Enhanced Magnetic Resonance Imaging
c-TACE	Conventional Transarterial Chemoembolization
CEUS	Contrast-Enhanced Ultrasound
CIN	Contrast-Induced Nephropathy
CT	Computed Tomography
DCE-US	Dynamic Contrast-Enhanced Ultrasound
DEB-TACE	Drug-Eluting Bead Transarterial Chemoembolization
DSA	Digital Subtraction Angiography
DWI	Diffusion-Weighted Imaging
FPS	Frames Per Second
HCC	Hepatocellular Carcinoma
HBV	Hepatitis B Virus
HCV	Hepatitis C Virus
IA-CEUS	Intra-Arterial Contrast-Enhanced Ultrasound
LI-RADS	Liver Imaging Reporting and Data System
MASLD	Metabolic dysfunction-associated Steatotic Liver Disease
MHz	Megahertz
MI	Mechanical Index
mRECIST	Modified Response Evaluation Criteria in Solid Tumors
MRI	Magnetic Resonance Imaging
mTT	Mean Transit Time
NSF	Nephrogenic Systemic Fibrosis
PE	Peak Enhancement
PES	Postembolization Syndrome
RCT	Randomized Controlled Trial
RT	Rise Time
SMA	Superior Mesenteric Artery
T2WI	T2-Weighted Imaging
TACE	Transarterial Chemoembolization
TIC	Time-Intensity Curve
TRA	Transarterial
TTP	Time to Peak
WiR	Wash-in Rate
3D-CEUS	Three-Dimensional Contrast-Enhanced Ultrasound
